# The treatment effect of rehabilitation gymnastics on postoperative rehabilitation in elderly patients with lumbar spinal stenosis

**DOI:** 10.3389/fmed.2025.1584965

**Published:** 2025-05-19

**Authors:** Yushan Guo, Xianghui Wang, Yingtao Shi

**Affiliations:** ^1^Operating Room, North China Medical and Health Group Xingtai General Hospital, Xingtai, China; ^2^Department of Nursing, North China Medical and Health Group Xingtai General Hospital, Xingtai, China

**Keywords:** spinal stenosis, rehabilitation gymnastics, compression treatment, prognosis, fusion surgery

## Abstract

**Objective:**

This study sought to evaluate the impact of integrating rehabilitation gymnastics with compression therapy on postoperative recovery outcomes in elderly patients undergoing surgery for lumbar spinal stenosis.

**Methods:**

Patients aged ≥65 years hospitalized for lumbar spinal stenosis between July 2016 and July 2018 were included. Eighty-four patients receiving postoperative rehabilitation gymnastics and compression therapy were assigned to the intervention group, while 84 matched controls received standard care alone. Both groups underwent routine postoperative management. Clinical outcomes were assessed at 2 weeks, 1 month, and 3 months post-surgery using measures including lower-extremity deep venous thrombosis (DVT) incidence, visual analog scale (VAS) pain scores, Oswestry Disability Index (ODI), Japanese Orthopaedic Association (JOA) scores, and patient satisfaction rates.

**Results:**

The intervention group demonstrated significantly reduced DVT incidence, lower VAS and ODI scores, and higher JOA scores and satisfaction rates at 2 weeks and 1 month post-surgery compared to controls (all *p* < 0.05). By the third month, however, no statistically significant differences were observed between the groups.

**Conclusion:**

Combining rehabilitation gymnastics with compression therapy enhances early postoperative recovery and functional outcomes in elderly lumbar spinal stenosis patients, though these benefits diminish by 3 months. These findings highlight the short-term efficacy of integrated rehabilitation strategies in accelerating postoperative healing.

## Introduction

Lumbar spinal stenosis (LSS), an important cause of chronic lower back pain, significantly impairs mobility and diminishes quality of life in affected individuals (1). Surgical intervention, particularly posterior lumbar fusion, is frequently employed to address spinal stenosis, with evidence supporting its superior efficacy over conservative treatments (2–8). While surgical precision remains a primary focus, postoperative complications, including lower-limb deep venous thrombosis (DVT), pose persistent challenges, particularly in elderly patients. Advanced age and prolonged postoperative bed rest, necessitated by degenerative conditions, heighten vulnerability to complications such as DVT, muscle atrophy, and nerve adhesions. Despite this elevated risk, research on optimizing recovery strategies for elderly LSS patients remains limited.

Current clinical guidelines advocate postoperative lower-limb rehabilitation exercises to mitigate complications. However, adherence to such regimens is often low among elderly patients due to discomfort and pain following surgery. To address this barrier, our study integrates compression therapy—a modality designed to enhance muscular contraction and relaxation, thereby improving circulation with structured rehabilitation gymnastics.

This investigation evaluates whether combining lower-limb rehabilitation gymnastics with compression therapy yields superior postoperative outcomes compared to conventional care in elderly LSS patients, focusing on functional recovery, complication prevention, and patient compliance.

## Materials and methods

### Ethics statement

This study was approved by the Ethics committee of North China Medical and Health Group Xingtai General Hospital. This is a retrospective case–control study in which we collected data and analyzed them anonymously without any potential harm to the patients. Thus, informed consent was waived by the ethics committee, given the retrospective design.

### Patients and inclusion criteria

**Figure 1:**
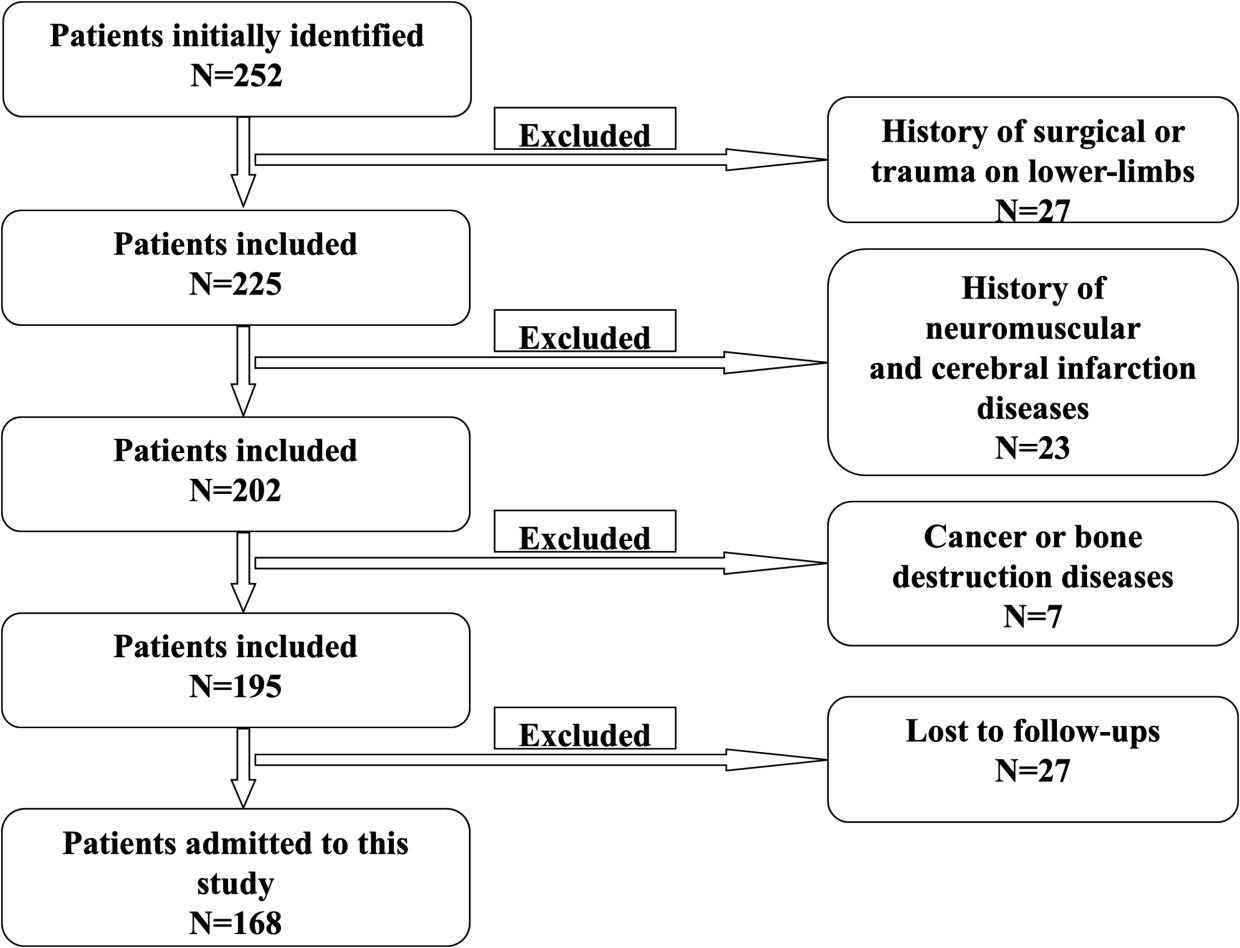
Scheme of patient selection.

**Figure 2:**
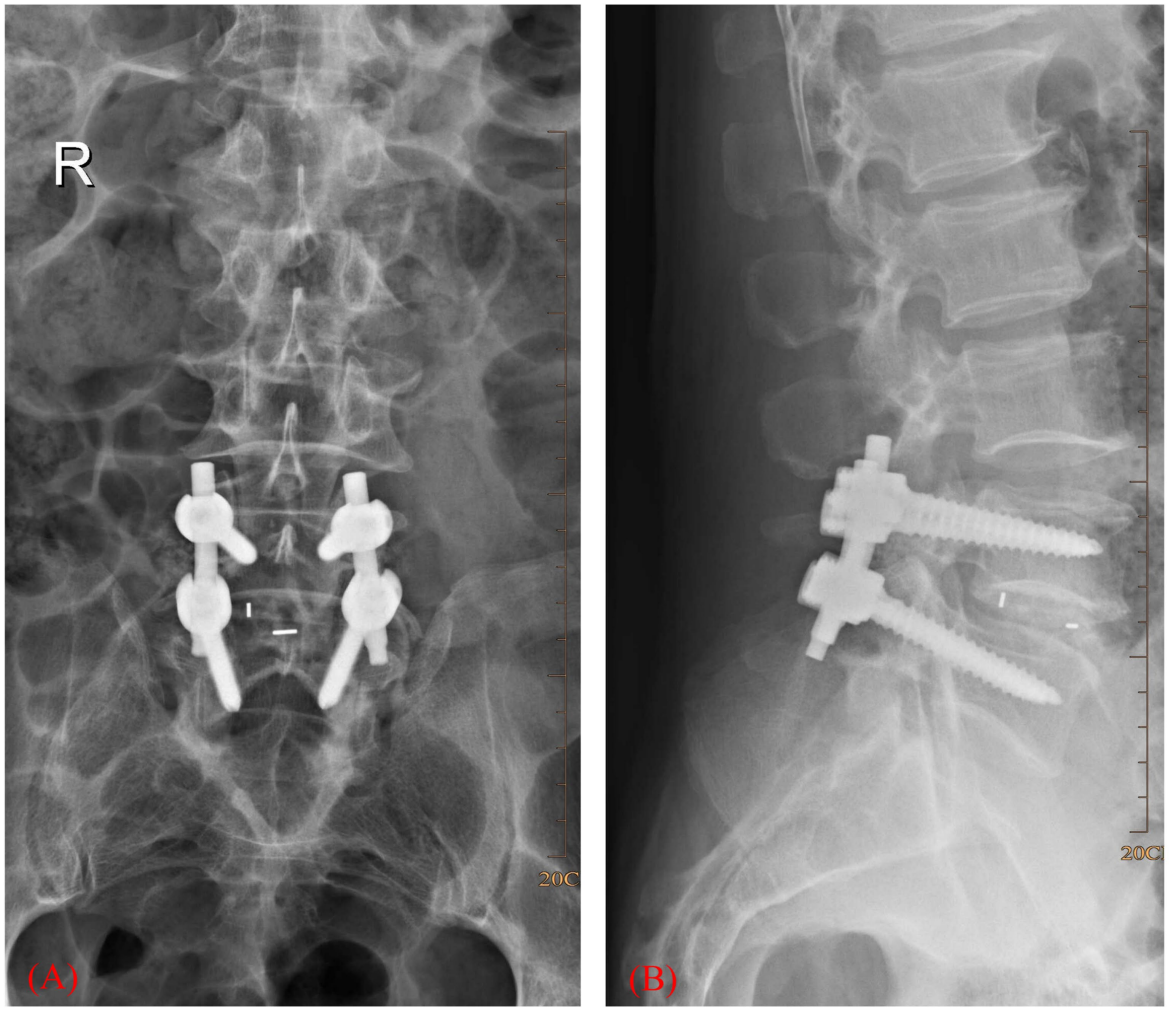
X-ray image showing lumbar interbody fusion surgery. **(A)** Coronary plane. **(B)** Sagittal plane.

Medical records of elderly patients (aged ≥65 years) who underwent surgery because of lumbar spinal stenosis between July 2016 and July 2018 were collected. Figure 1 is a scheme showing the process of enrolling the patients in our study. Finally, 168 patients were recruited in our study, 84 in the intervention group and another 84 in the control group. There were 103 males and 65 females. The age was 68 ± 3 years on average. Figure 2 shows a postoperative X-ray image of the spine after the lumbar fusion surgery. The inclusion criteria for the patients in our study are listed as follows. 1. Patients underwent lumbar spinal fusion surgery; 2. no history of trauma or operation on the lower limbs; 3. no neuromuscular or cerebral infarction disease history; 4. no cancer or bone destruction diseases; 5. muscle strength of preoperative lower limbs was grade IV or grade V; 6. no preoperative lower-limb DVT; 7. no infection after surgery; 8. no nerve injury was observed. Patients that did not apply to these criteria were excluded from this study.

### Group allocation and demographics

The cohort was divided into two age-matched groups (*n* = 84 each): an intervention group (54 males, 32 females) and a control group (47 males, 39 females). Both groups received standardized perioperative care, including routine nursing protocols, pharmacological management, and postoperative administration of low-molecular-weight heparin.

### Intervention protocols

The intervention group underwent a 3-month regimen combining lower-limb rehabilitation exercises with compression therapy, while the control group received standard care alone. The rehabilitation exercise protocol was performed as follows (9).

1. Initial Relaxation: Patients assumed a supine position for muscle relaxation.
2. Lower-Limb Massage: A 5-min centripetal massage was administered to both limbs.
3. Ankle Pump Exercise: Repeated flexion-extension of the ankles (5 s per movement, 50 repetitions/set, 3 daily sets).
4. Knee-Pressing Exercise: Legs were fully extended while pressing knees downward (10-s holds, 20 repetitions/set, 3 daily sets).
5. Straight Leg Raises: Active or passive elevation of lower limbs (30 repetitions/leg, 5 daily sets).
6. Hip/Knee Flexion: Patients bent knees and hips to 30° flexion in a relaxed state (20 repetitions/set, 3 daily sets).

The POWER Q-1000 pneumatic device delivered sequential compression via air-filled leggings covering the lower limbs and thigh base. Parameters included: (1) limb compression: 40 mmHg pressure, 10 mmHg incremental steps, 8-s inflation intervals; (2) foot compression: 130 mmHg pressure, 10 mmHg steps, 5-s inflation intervals. Sessions lasted 30 min twice daily.

### Follow-up and outcome measures

Postoperative assessments were conducted at 2 weeks, 1 month, and 3 months. Outcomes included: (1) patient satisfaction: stratified as “very satisfied,” “satisfied,” or “dissatisfied”; (2) clinical metrics: incidence of lower-limb DVT, Visual Analog Scale (VAS) pain scores, Oswestry Disability Index (ODI), and Japanese Orthopaedic Association (JOA) functional scores.

### Statistical analyses

All statistical analyses were performed using SPSS 18.0 (IBM SPSS Inc., United States). Continuous variables were assessed for normality using the Shapiro–Wilk test (*p* > 0.10). Normally distributed data are presented as mean ± standard deviation (SD); non-normally distributed data are expressed as median (interquartile range, IQR). Parametric comparisons between groups were conducted using Student’s *t*-test for data meeting assumptions of normality and homogeneity of variance (Levene’s test). Non-parametric analyses employed the Mann–Whitney *U* test. Categorical variables—including deep venous thrombosis (DVT) incidence and patient satisfaction rates—were evaluated using Pearson’s chi-square test. A two-tailed *p*-value < 0.05 defined statistical significance.

## Results

### Lower limb DVT

As indicated in Table 1, two weeks after surgery, the incidence of lower limb DVT in the intervention group was lower than in the control group (χ^2^ = 3.982, *p* = 0.046). Similarly, at one-month post-surgery, the intervention group had a lower incidence of DVT compared to the control group (χ^2^ = 4.085, *p* = 0.043). However, by the third month, no significant difference in lower limb DVT was observed between the two groups (χ^2^ = 2.39, *p* = 0.122).

**Table 1:** Comparison of intervention group with control group regarding lower limb deep venous thrombosis.

After surgery	Intervention (*n* = 84)	Control (*n* = 84)	Chi-square test	
	Non-DVT/ DVT	Non-DVT/ DVT	χ^2^	*P*
2 weeks	79 cases/5 cases	71 cases/13 cases	3.98	*0.046*
1 month	81 cases/3 cases	74 cases/10 cases	4.09	*0.043*
3 months	83 cases/1 cases	78 cases/6 cases	2.39	0.122

DVT, deep venous thrombosis. Italic values indicate statistical significance (*P* < 0.05).

### VAS score

Table 2 shows that the VAS scores were significantly lower in the intervention group than in the control group at both the second week and first month after surgery (*t* = 8.11, *p* < 0.001; *t* = 8.64, *p* < 0.001, respectively). However, no significant difference in VAS scores was found between the groups at the third month post-surgery (*t* = 1.05, *p* > 0.295).

**Table 2:** Comparison of intervention group with control group regarding the VAS score.

After surgery	Intervention (*n* = 84)	Control (*n* = 84)	Student’s *t*-test	
	Mean± SD	Mean± SD	*t*	*P*
2 weeks	3.32 ± 1.61	5.10 ± 1.21	8.11	*<0.001*
1 month	2.01 ± 0.95	3.40 ± 1.13	8.64	*<0.001*
3 months	1.45 ± 0.70	1.57 ± 0.76	1.05	0.295

VAS, visual analog scale; SD, standard deviation. Italic values indicate statistical significance (*P* < 0.05).

### ODI

As presented in Table 3, the ODI scores were lower in the intervention group than in the control group during the second week and first month after surgery (*t* = 6.35, *p* < 0.001; *t* = 6.16, *p* < 0.001, respectively). Nevertheless, by the third month, there was no significant difference in ODI scores between the intervention and control groups (*t* = 1.00, *p* = 0.314).

**Table 3:** Comparison of intervention group with control group regarding the ODI.

After surgery	Intervention (*n* = 84)	Control (*n* = 84)	Student’s *t* test	
	Mean± SD	Mean± SD	*t*	*P*
2 weeks	50.26 ± 3.79	55.98 ± 7.32	6.35	*<0.001*
1 month	30.32 ± 3.46	35.64 ± 7.12	6.16	*<0.001*
3 months	21.47 ± 3.20	21.94 ± 2.74	1.00	0.314

ODI, Oswestry Disability Index; SD, standard deviation. Italic values indicate statistical significance (*P* < 0.05).

### JOA score

As shown in Table 4, two weeks and 1 month after surgery, the JOA score was higher in the intervention group compared to the control group (*t* = −7.40, *p* < 0.001; *t* = −6.12, *p* < 0.001, respectively). However, by the third month post-surgery, no significant difference in JOA scores was observed between the intervention and control groups (*t* = −1.454, *p* = 0.148).

**Table 4:** Comparison of intervention group with control group regarding the JOA score.

After surgery	Intervention (*n* = 84)	Control (*n* = 84)	Student’s *t*-test	
	Mean± SD	Mean± SD	*t*	*P*
2 weeks	14.21 ± 2.97	10.19 ± 4.00	−7.40	*<0.001*
1 month	19.65 ± 3.09	16.81 ± 2.94	−6.12	*<0.001*
3 months	25.57 ± 1.73	25.17 ± 1.87	−1.45	0.148

JOA, Japanese Orthopaedic Association; SD, standard deviation. Italic values indicate statistical significance (*P* < 0.05).

### Satisfaction survey

According to Table 5, the satisfaction rate was higher in the intervention group compared to the control group at both the two-week and one-month marks after surgery (χ^2^ = 9.052, *p* = 0.011; χ^2^ = 7.187, *p* = 0.027, respectively). However, by the third month, no significant difference in satisfaction rates was observed between the two groups (χ^2^ = 2.106, *p* = 0.349).

**Table 5:** Comparison of intervention group with control group regarding satisfaction survey.

After surgery	Intervention (*n* = 84)	Control (*n* = 84)	Chi-square test	
	Very satisfied/satisfied/dissatisfied	Very satisfied/satisfied/dissatisfied	χ^2^	*P*
2 weeks	36 cases/38 cases/10 cases	28 cases/30 cases/26 cases	9.052	*0.011*
1 month	47 cases/33 cases/4 cases	41 cases/28 cases/15 cases	7.187	*0.027*
3 months	52 cases/30 cases/2 cases	49 cases/29 cases/6 cases	2.106	0.349

Italic values indicate statistical significance (*P* < 0.05).

## Discussion

Lumbar spinal stenosis (LSS) in elderly patients is primarily attributed to degenerative pathologies such as lumbar disc herniation, ligamentum flavum hypertrophy, vertebral osteophytes, and spondylolisthesis (1). These conditions frequently result in debilitating symptoms, including chronic lower back pain and functional limitations, which markedly impair mobility and quality of life (2). Surgical decompression, often involving the removal of osteophytes, hypertrophic ligaments, and degenerative tissues, remains a standard intervention to alleviate neural compression (3–5).

In this study, we evaluated the efficacy of combining rehabilitation gymnastics with compression therapy to enhance postoperative recovery in elderly LSS patients. Compared to controls receiving standard care, the intervention group demonstrated superior short-term outcomes, including reduced lower-limb DVT incidence, VAS pain scores, improved ODI and JOA scores, and higher patient satisfaction at 2 weeks and 1 month postoperatively (all *p* < 0.05). However, these differences were no longer statistically significant by the third month. While both groups achieved comparable long-term recovery, the intervention group exhibited accelerated functional restoration during the critical early postoperative phase (e.g., reduced hospital stays, cost-effectiveness) despite transient effects.

The rising global prevalence of spinal surgery, particularly among aging populations, underscores the need for optimized rehabilitation strategies (10–13). LSS is now the leading indication for spinal surgery in elderly patients (3), with common techniques including decompressive laminectomy and fusion (14). Despite procedural advancements, functional recovery remains inconsistent, with studies reporting wide variability in success rates: patient satisfaction ranges from 15 to 81% (15–17), and functional improvement spans 58–69% (13, 18, 19). Postoperative complications, such as trunk muscle dysfunction, further complicate recovery (20), highlighting the importance of structured rehabilitation.

The clinical utility of postoperative rehabilitation, however, remains debated. Prior research indicates that home-based physiotherapy programs, including strength and flexibility exercises, fail to improve long-term functional outcomes (21). Similarly, rehabilitation protocols and educational interventions show limited impact on postoperative management in extended follow-ups (22). A randomized controlled trial with 1-year follow-up found no significant benefit from active rehabilitation in spinal fusion patients (23). These discrepancies may stem from non-standardized protocols and poor patient adherence, often due to postoperative pain. In contrast, our integrated approach—combining guided rehabilitation gymnastics to mitigate muscle atrophy with compression therapy to enhance circulation, addressed these barriers, yielding measurable early benefits over conventional care. Our findings show that rehabilitation gymnastics combined with compression treatment is effective for facilitating recovery after lumbar spinal surgery. Thus, we would suggest that this method of postoperative rehabilitation should be widely promoted in the clinic.

There are some limitations in this study. Firstly, it is difficult to avoid selection bias when selecting cases because of the retrospective study. Secondly, recall bias is difficult to avoid when obtaining medical record information. At last, considering the small sample size of only 168 patients from a single-center and the homogeneous demographics (e.g., age 68 ± 3 years), this may affect the generalizability of the findings and limit the applicability of this study to diverse populations. Thus, future multi-center studies with larger sample sizes are warranted to validate our findings.

In conclusion, our findings suggest that rehabilitation gymnastics paired with compression therapy accelerates early postoperative recovery in elderly LSS patients undergoing posterior lumbar fusion. This non-invasive strategy offers a pragmatic solution to enhance short-term outcomes, though its effects attenuate over time. Given the challenges of postoperative adherence and variable surgical success rates, this combined regimen represents a valuable adjunct to standard care. Future studies should explore protocol refinements to sustain long-term efficacy.

## Data Availability

The raw data supporting the conclusions of this article will be made available by the authors, without undue reservation.

## References

[1] Atlas SJ Keller RB Wu YA Deyo RA Singer DE. Long-term outcomes of surgical and nonsurgical management of sciatica secondary to a lumbar disc herniation: 10 year results from the Maine lumbar spine study. Spine. (2005) 30:927–35. doi: 10.1097/01.brs.0000158954.68522.2a 15834338

[2] Aalto TJ Leinonen V Herno A Alen M Kröger H Turunen V. Postoperative rehabilitation does not improve functional outcome in lumbar spinal stenosis: a prospective study with 2-year postoperative follow-up. Eur Spine J. (2011) 20:1331–40. doi: 10.1007/s00586-011-1781-y, PMID: 21523459 PMC3175851

[3] Huo Y Ding W Rudd S Yang D Ma L Zhao R. Incidence and risk factors of lumbar plexus injury in patients undergoing oblique lumbar interbody fusion surgery. Eur Spine J. (2023) 32:336–44. doi: 10.1007/s00586-022-07439-w, PMID: 36370208

[4] Chou R Baisden J Carragee EJ Resnick DK Shaffer WO Loeser JD. Surgery for low back pain: a review of the evidence for an American pain society clinical practice guideline. Spine. (1976) 2009:1094–109. doi: 10.1097/BRS.0b013e3181a105fc 19363455

[5] Li Z Gao X Ding W Li R Yang S. Asymmetric distribution of Modic changes in patients with lumbar disc herniation. Eur Spine J. (2023) 32:1741–50. doi: 10.1007/s00586-023-07664-x, PMID: 36977942

[6] Deyo RA Gray DT Kreuter W Mirza S Martin BI. United States trends in lumbar fusion surgery for degenerative conditions. Spine. (1976) 30:1441–5. doi: 10.1097/01.brs.0000166503.37969.8a 15959375

[7] Deyo RA Mirza SK Martin BI Kreuter W Goodman DC Jarvik JG. Trends, major medical complications, and charges associated with surgery for lumbar spinal stenosis in older adults. JAMA. (2010) 303:1259–65. doi: 10.1001/jama.2010.338, PMID: 20371784 PMC2885954

[8] Du Bois M Szpalski M Donceel P. A decade’s experience in lumbar spine surgery in Belgium: sickness fund beneficiaries, 2000-2009. Eur Spine J. (2012) 21:2693–703. doi: 10.1007/s00586-012-2381-1, PMID: 22661202 PMC3508248

[9] Wang H Huo Y Zhao Y Zhang B Yang D Yang S. Clinical rehabilitation effect of postoperative lower-limb training on the patients undergoing OLIF surgery: a retrospective study. Pain Res Manag. (2020) 2020:1–6. doi: 10.1155/2020/1065202, PMID: 32015783 PMC6985932

[10] Gunzburg R Szpalski M. The conservative surgical treatment of lumbar spinal stenosis in the elderly. Eur Spine J. (2003) 12:S176–80. doi: 10.1007/s00586-003-0611-2, PMID: 12961080 PMC3591835

[11] Gibson JNA Waddell G. Surgery for degenerative lumbar spondylosis: an updated Cochrane review. Spine. (2005) 30:2312–20. doi: 10.1097/01.brs.0000182315.88558.9c, PMID: 16227895

[12] Tian X Zhao H Yang S Ding W. The effect of diabetes mellitus on lumbar disc degeneration: an MRI-based study. Eur Spine J. (2024) 33:1999–2006. doi: 10.1007/s00586-024-08150-8, PMID: 38361008

[13] Ilves O Häkkinen A Dekker J Pekkanen L Piitulainen K Järvenpää S. Quality of life and disability: can they be improved by active postoperative rehabilitation after spinal fusion surgery in patients with spondylolisthesis? A randomised controlled trial with 12-month follow-up. Eur Spine J. (2017) 26:777–84. doi: 10.1007/s00586-016-4789-5, PMID: 27687823

[14] Jansson KA Blomqvist P Granath F Németh G. Spinal stenosis surgery in Sweden 1987-1999. Eur Spine J. (2003) 12:535–41. doi: 10.1007/s00586-003-0544-9 12768381 PMC3468016

[15] McGregor AH Doré CJ Morris TP Morris S Jamrozik K. ISSLS prize winner: function after spinal treatment, exercise, and rehabilitation (FASTER): a factorial randomized trial to determine whether the functional outcome of spinal surgery can be improved. Spine. (2011) 36:1711–20. doi: 10.1097/BRS.0b013e318214e3e6 21378603

[16] McGregor AH Hughes SP. The evaluation of the surgical management of nerve root compression in patients with low back pain: part 1: the assessment of outcome. Spine. (1976) 27:1465–70. doi: 10.1097/00007632-200207010-00018 12131748

[17] Malmivaara A Slatis P Heliovaara M Sainio P Kinnunen H Kankare J. Surgical or nonoperative treatment for lumbar spinal stenosis? A randomized controlled trial. Spine. (1976) 32:1–8. doi: 10.1097/01.brs.0000251014.81875.6d 17202885

[18] Macfarlane GJ Thomas E Croft PR Papageorgiou AC Jayson MIV Silman AJ. Predictors of early improvement in low back pain amongst consulters to general practice: the influence of pre-morbid and episode-related factors. Pain. (1999) 80:113–9. doi: 10.1016/S0304-3959(98)00209-7, PMID: 10204723

[19] Rhee JM Schaufele M Abdu WA. Radiculopathy and the herniated lumbar disc. Controversies regarding pathophysiology and management. J Bone Joint Surg. (2006) 88:2069–80. doi: 10.2106/00004623-200609000-00023 17036418

[20] Stromqvist B Jonsson B Fritzell P Hägg O Larsson BE Lind B. The Swedish National Register for lumbar spine surgery: Swedish society for spinal surgery. Acta Orthop Scand. (2001) 72:99–106. doi: 10.1080/000164701317323327, PMID: 11372956

[21] Turner JA Ersek M Herron L Deyo R. Surgery for lumbar spinal stenosis. Attempted meta-analysis of the literature. Spine. (1976) 17:1–8. doi: 10.1097/00007632-199201000-00001, PMID: 1531550

[22] Weinstein JN Tosteson TD Lurie JD Tosteson ANA Blood E Hanscom B. Surgical versus nonsurgical therapy for lumbar spinal stenosis. N Engl J Med. (2008) 358:794–810. doi: 10.1056/NEJMoa0707136, PMID: 18287602 PMC2576513

[23] Yee A Adjei N Do J Ford M Finkelstein J. Do patient expectations of spinal surgery relate to functional outcome? Clin Orthop Relat Res. (2008) 466:1154–61. doi: 10.1007/s11999-008-0194-7, PMID: 18347892 PMC2311462

